# Effect of Organic Manure and Moisture Regimes on Soil Physiochemical Properties, Microbial Biomass C_mic_:N_mic_:P_mic_ Turnover and Yield of Mustard Grains in Arid Climate

**DOI:** 10.3390/plants11060722

**Published:** 2022-03-08

**Authors:** Rajendra Bhanwaria, Bikarma Singh, Carmelo Maria Musarella

**Affiliations:** 1 Academy of Scientific and Innovative Research (AcSIR), Ghaziabad 201002, Uttar Pradesh, India; rbhanwaria@iiim.res.in; 2 Plant Sciences and Agrotechnology Division, CSIR-Indian Institute of Integrative Medicine, Jammu 180001, Jammu & Kashmir, India; 3 Botanic Garden Division, CSIR-National Botanical Research Institute, Rana Pratap Marg, Lucknow 226001, Uttar Pradesh, India; 4 Department of Agraria, Mediterranea University of Reggio Calabria, Feo di Vito snc, 89122 Reggio Calabria, Italy; carmelo.musarella@unirc.it

**Keywords:** arid condition, C:N:P, organic fertilizer, soil water regimes, mustard, salinity, soil microbial biomass

## Abstract

(1) Background: Arid conditions occur due to climate abnormality in the different biogeography regions of the world. The aim of this research is to investigate the stoichiometry of manure and moisture regimes on soil properties, microbial biomass C:N:P turnover, and the grain yield of mustard crops under stress in arid conditions; (2) Methods: The field experiment was carried out for 2 years at the farms of the agriculture college of SKN, Jobner (SKRAU Bikaner, Rajasthan). The effects of organic manure, moisture regimes, and saline water treatment on soil properties, such as the soil microbial biomass build-up, loss, turnover, and recycling of carbon (C_mic_), nitrogen (N_mic_), and phosphorus (P_mic_) in the mustard crop were investigated. The twenty-seven treatments studied are described as follows: organic manures (control, FYM @ 10 t ha^−1^ and vermicompost @ 5 t ha^−1^), moisture regimes (0.4, 0.6, and 0.8 IW/CPE ratio), and saline irrigation water (control, 6, 12 dSm^−1^); (3) Results: Our findings indicate that vermicompost @ 5 t ha^−1^ significantly increases moisture retention and the available water in soil at 33 kPa and 1500 kPa. The microbial biomass build-up of C_mic_ increases by 43.13% over the control and 14.36% over the FYM. Similarly, the soil microbial biomass of N_mic,_ and P_mic_ also increase considerably. The SHC of the soil is enhanced by the application of farmyard fertilizer and vermicompost. The BD and pH decrease significantly, while the SHC, OC, CEC, and ECe of the soil increase significantly. The build-up, losses, and fluxes of the soil microbial biomass of C_mic_, N_mic_, and P_mic_ increase significantly, and the turnover rate decreases under vermicompost @ 5 t ha^−1^. A significant increase in grain yield was observed. Irrigation with a 0.8 IW/CPE moisture regime significantly decreases the pH of the SHC; (4) Conclusions: We hypothesized the interactive outcomes of the moisture regime and found that organic manure significantly influenced grain and stover yield. The treatments of quality irrigation water and the addition of organic manure are efficient enough to improve soil properties, water holding capacity, and soil microbial biomass C:N:P in stress climatic conditions.

## 1. Introduction

Plants show specific thresholds of soil accumulation to improve their performance and adapt to a given environment [1,2]. Salinity and element availability differentially affect growth, leaf morphology, water relations, solutes accumulation, and antioxidant capacity in plants [3,4]. It has been observed that the constant changes in the fertility of the soil occurred through various organic amendment practices [5]. Soil organic matter (SOM), characterized by the residence time and biodiversity present, regulates the movement of water by increasing the moisture-retaining capacity and provides essential nutrients for plants and microbes [6]. On the contrary, anthropic intervention with herbicides causes a decrease in the water content of the soil, which is faster in areas with less vegetation [7]. SOM also improves and controls the stability of the soil structure as well as the number of essential aggregates [8]. Nutrient cycling is an essential component to sustain agricultural productivity concerning soil–microbe interaction, and the population of microbes in soil plays a key function in organic matter turnover [9,10]. The use of organic manure has a significant role in improving soil biological, physical, and chemical characteristics [11], and the application of organic fertilizers maintains the crop productivity and soil nature [12] and is responsible for high biodiversity [13]. Nitrogen and organic carbon are the major part of the organic content at the upper layer of soil under grazing and pasture land [14,15]. Soil microorganisms and their biomass play a pivotal part in the development of the organic composition of soil and the availability of essential aggregates in the soil for agricultural purposes [16]. They are the most important components of C:N:P, responsible for the mobilization of the available nutrients to the plant, their uptake, mineralization, and microbial biomass turnover [17]. The formation of microbial biomass and turnover through the process of organic matter decomposition differs in the various parameters of soil, such as soil texture, land-use pattern, and other changes [18,19]. The growth of microorganisms can be stimulated through the presence of enough carbon substrates; however, the death of microbes usually releases the carbon, nitrogen, and phosphorus into bio-available carbon, nitrogen, and phosphorus pools [20] to be reused by a new generation microbes and plants for their growth and life cycles [21,22]. The nutrients obtained from natural organic matters help to improve soil health and retain the productivity of crops in harsh climatic zones. Microbes occurring in the soil serve as the flow of energy that leads to the transformation of the pool of the essential substances C:N:P, and the soil fertility depends on the rate of turnover of organic substances brought with the different activities of microbes.

The oil grain production sector occupies the best global rank in India in terms of the agricultural economy and is ranked second at a global level in terms of production (6.82 Mt). *Brassica juncea* (L.) Czern. (Brassicaceae) and Indian mustard (synonym: *Sinapis juncea* L.) are some of the most prominent rabi crops for millions of people, and demand for Indian oil gains is increasing day by day. Rajasthan state is situated near the Great Indian Thar Desert in the north-western part of India and is susceptible to a loss of good quality water availability, causing the harsh impact of climate change. This state produces one-third of the total oil grains in India [23]. Approximately 80% of global mustard cultivation is undertaken in semi-arid and arid climatic conditions where the quality of groundwater is very poor and crop cultivation is dependent on monsoon rain or the early irrigation of the growing season. The water used for irrigation is of poor quality and creates soil saline or sodicity in agricultural land, which affects the significant areas of fertile tracts and causes significant reductions to crop production and soil productivity.

The aim of this study was to investigate the stoichiometry of manure and moisture regimes on soil properties, microbial biomass C:N:P, and the grain yield of a mustard crop under stress arid conditions. The objectives were to: (i) undertake a split-plot experiment with mustard by using treatments that combine bio-based manures (farmyard manure and vermicompost), moisture regimes, and saline irrigation water in the arid climate of Rajasthan; (ii) carry out an in-depth characterization of the physico-chemical properties of arid soil suitable for the mustard crop; (iii) investigate the effects of organic manures, moisture regimes, and saline water treatment for the microbial build-up, loss, turnover, and recycling of carbon (C_mic_), nitrogen (N_mic_), and phosphorus (P_mic_) for the grain yield and stover yield for mustard in stress condition; and (iv) propose a methodology to select the most suitable organic amendments and deepen the knowledge of the potential benefits associated with the application of composted organic manures. We hypothesized that: (i) organic manures and moisture regimes enhance the population of microorganisms and crop production; (ii) soil biological activity such as the microbial biomass of C_mic_, N_mic,_ and P_mic_, and their build-up, losses, fluxes, and turn-over rates reduces due to the increasing level of saline water; (iii) the interactive effect of the different levels of moisture regimes and treatment using farmyard manure and vermicompost influences grain and stover yield, which is due to an increase in saturated hydraulic conductivity and ECe, and the in-process pH, CEC, and organic carbon content decrease significantly; and (iv) the use of bio-based fertilizers enhances the soil nutrients and their recycling potential to enhance carbon, nitrogen, and phosphorus, and other available nutrients.

## 2. Results

### 2.1. Average Weekly Weather Parameters

**Figure 1:**
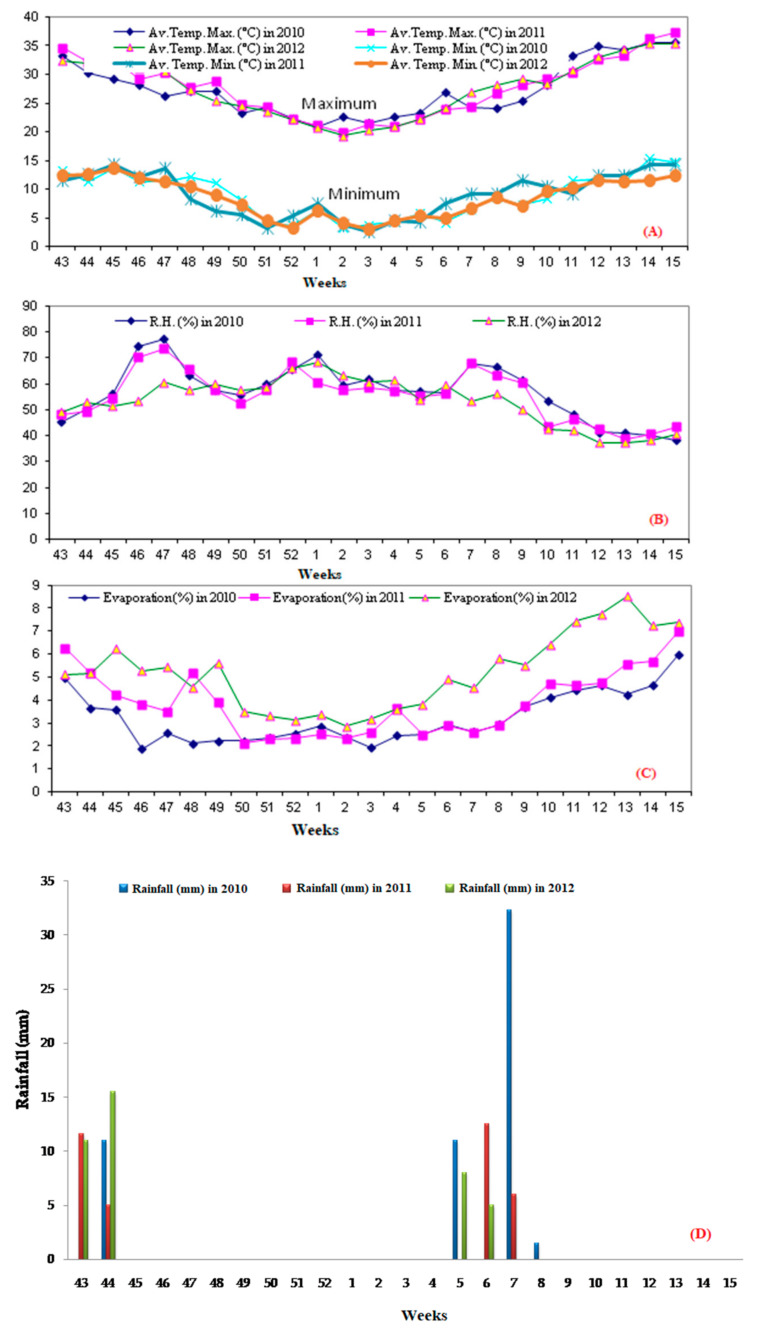
(**A**–**D**). Weekly weather parameters for consecutive three years for rabi crop cultivation in an arid region (Rajasthan state) of India: (**A**): average temperature, *X*-axis represents metrological weeks, *Y*-axis represents the mean temperature in degree centigrade; (**B**): average relative humidity, *X*-axis represents metrological weeks, *Y*-axis represents mean relative humidity in percentage; (**C**): evaporation, *X*-axis represents metrological weeks, *Y*-axis represents mean evaporation in percentage; (**D**): annual rainfall, *X*-axis represents metrological weeks, *Y*-axis represents mean annual rainfall in millimeters.

During the experimental periods, the weekly average maximum temperature recorded was in the 15th week of all the three consecutive years, i.e., 35.57 °C (2010), 37.28 °C (2011), and 35.25 °C (2012), respectively, while the minimum weekly average temperature was 3.29 °C in the 2nd week of 2010, 2.67 °C in the 3rd week of 2011, and 3.07 °C in the 3rd week of 2012 (Figure 1A). The highest relative humidity was 77.33% in 2010, 73.33% in 2011, and 68.22% in 2012. This was recorded in the 47th week and the 1st week in every years, respectively. The lowest relative humidity recorded was 38.39% in the 15th week, 38.87% in 12th week, and 37.27% also in the 12th week of the consecutive years, respectively (Figure 1B). The evaporation was recorded to be a maximum or minimum of 5.98% in the 15th or 1.89% in the 46th week of 2010, 6.9% in 15th or 2.11% in the 50th week of 2011, and 8.52% in 13th or 2.85% in the 2nd week of 2012, respectively (Figure 1C). The maximum amount of annual rainfall recorded during 2010, 2011, and 2012 was 32.3 mm in the 7th week, 12.5 mm in the 6th week, and 15.5 mm in the 44th week, respectively (Figure 1D).

### 2.2. Soil Physico-Chemical Properties at Harvest

#### 2.2.1. Bulk Density (BD)

The bulk density decreased with respect to the organic parameters over control (M_o_). After the application of the vermicompost (M_2_) treatment, the highest reductions were observed in bulk density followed by farmyard manure (M_1_). It was explicit from the data via the application of several levels of moisture regimes that the bulk density of the soil did not have any significant influence, but it showed the advancement of the trend. Similarly, the bulk density of the soil did not have any significant influence on the increasing level of saline water, but there were decreasing trends observed because of irrigation with different saline waters in the pooled mean (Table 1).

#### 2.2.2. Saturated Hydraulic Conductivity (SHC)

The saturated hydraulic conductivity (SHC) of the soil was enhanced by the application of farmyard fertilizers and vermicompost above the control. The SHC observed was higher under the application of the farmyard manure, and was 9.14% and 29.11% higher compared to the vermicompost and control, respectively. The SHC of the soil decreased significantly with the increased levels of moisture regimes over an I_1_(0.4 IW/CPE) level. The maximum pooled SHC of I_1_(0.4 IW/CPE) was observed to be higher in comparison to I_2_(0.6 IW/CPE) and I_3_(0.8 IW/CPE) by 8.06% and 11.64%, respectively. With the increasing level of salinity in water, 6 dSm^−1^ and 12 dSm^−1^, the SHC of the soil increased by 3.50% and 5.76%, respectively, which is significantly over the control (C_0_) (Table 1).

#### 2.2.3. Moisture Retention

The retention of soil moisture at 33 kPa and 1500 kPa, and available water content, increased significantly after the utilization of organic fertilizers. All the organic manures were equally efficient in increasing the soil moisture retention at 33 and 1500 kPa tensions. The application of vermicompost as a treatment in soil showed an enhancement in moisture capacity at 33 kPa (12.27%) and 1500 kPa (3.91%). Similarly, the water content presence (8.36%) was recorded highest with the same treatment. Further, the retention of soil moisture at 33 kPa and 1500 kPa, and available water content, increased with an enhanced level of moisture regimes or water frequency. The moisture regimes I_1_(0.4 IW/CPE) and I_2_(0.6 IW/CPE) remained equally effective compared to the retention of moisture at 33 kPa. The moisture regimes I_2_(0.6 IW/CPE) and I_3_(0.8 IW/CPE) were statistically on par with each other regarding moisture retention at 1500 kPa. The presence of water content in soil was significantly enhanced by enhancing the frequency of irrigation. The highest moisture retention was observed with the higher-level I_3_(0.8 IW/CPE) among the moisture regimes. Enhancing the salt concentration of the water significantly decreased the available water and moisture retention at 33kPa and 1500 kPa tensions. The maximum decrease was observed under C_2_(12 dSm^−1^), with higher levels over the C_0_(Control) and C_1_(6 dSm^−1^)in moisture retention at 33 kPa observed at 15.98% and 6.70%, respectively. The corresponding loss in moisture capacity at 1500 kPa was 15.04% and 4.46%, and the available water content decreased due to C_2_(12 dSm^−1^) and C_1_(6 dSm^−1^) by 16.40% and 7.81%, respectively.

#### 2.2.4. pH

The usages of different organic manures significantly decreased soil pH, but the application of FYM was observed as being on par in comparison to the control. The highest pH value recorded was 8.58 under the control, which was then reduced to 8.22% due to the utilization of FYM and 7.58% by the usage of vermicompost. The highest significant reduction of 9.71% was recorded due to the application of vermicompost, followed by a 4.38% reduction from the FYM over the control. The various levels of the moisture regimes significantly decreased the soil pH. The highest pH value of 8.83 in the soil was recorded under the I_1_(0.4 IW/CPE) level and was reduced to 8.12 and 7.67 due to the application of the I_2_(0.6 IW/CPE) and I_3_(0.8 IW/CPE) moisture regimes. The different concentrations of salinity varied significantly and different salinity levels decreased the soil pH at the crop harvest. The lowest value of 7.85 was observed under C_2_(12 dSm^−1^).

#### 2.2.5. Electrical Conductivity (ECe)

The organic manures significantly enhanced the soil ECe in the experimental site. The maximum ECe was enhanced due to the use of all the organic fertilizer over the control. The application of different levels of moisture regimes increased the ECe value at 2.58 dSm^−1^ of I_1_(0.4 IW/CPE) to 2.60 and 2.66 dSm^−1^ at the I_2_(0.6 IW/CPE) and I_3_(0.8 IW/CPE) moisture regimes, respectively. The enhancement of the salt concentration above the control, led to significant ECe production. The highest pooled ECe of the soil was observed under C_2_(12 dSm^−1^) and was lowest under the C_0_(control) treatment.

#### 2.2.6. Capacity of Cation Exchange (CEC)

The CEC of the soil significantly increased with the applications of all the organic manures above the control, where the usage of FYM was at 10 t ha^−1^. Being more with the control, the highest CEC was observed under the usage of vermicompost @ 5 t ha^−1^, which was 2.92% and 19.14% higher than the FYM and experimental control, respectively. The increased levels of the moisture regimes and the salt concentration of the water did not reveal any significant influence on the CEC of the soil, but an increasing trend was recorded due to the increased levels of the moisture regimes as well as the frequency of the irrigation water, while, there was a decreasing trend due to the usage of different levels of saline water.

#### 2.2.7. Organic Carbon (OC)

The organic fertilizers increased the organic carbon of soil (SOC) content above the control. The maximum organic carbon content observed in vermicompost was 28.50% higher over the control and 4.89% over the FYM. The utilization of irrigation in the I_1_(0.4 IW/CPE), I_2_(0.6 IW/CPE), and I_3_(0.8 IW/CPE) moisture level regimes significantly enhanced the soil OC. The maximum OC was noted under the I_3_ moisture regime, which were 5.65% and 6.11% higher than I_1_(0.4 IW/CPE) and I_2_(0.6 IW/CPE) moisture regimes, respectively. The usage of different levels of saline water significantly decreased the OC content in the soil. The drastic reduction of organic carbon was maximum under C_2_(12 dSm^−1^), which was observed to be 29% lower than C_1_(6 dSm^−1^). The application of C_1_(6 dSm^−1^) decreased the organic carbon by 22.50% compared to the control.

### 2.3. Soil Microbial Biomass

#### 2.3.1. Effect on Microbial Build-Up

Organic fertilizers increased the total microbial biomass of C_mic_, N_mic_, and P_mic_. The microbial biomass build-up was recorded to be significantly higher under vermicompost treatment, whereas, the lowest was observed under the control condition. This increased the build-up of C_mic_ by 43.13% compared to the control and 14.36% compared to the FYM. Similarly, there was an increase in N_mic_ because of vermicompost by 37.43% above the control and an increase of 8.48% above the FYM. The consequent increase in P_mic_ due to vermicompost was 12.67% and 37.10% over the FYM and control, respectively. Besides, it was observed that the use of irrigation at several levels of moisture regimes increased the total of the microbial biomass of C_mic_, N_mic_, and P_mic_. It was recorded that the build-up was considerably highest under the I_3_(0.8 IW/CPE) moisture regime, whereas, the lowest was recorded under I_1_(0.4 IW/CPE). The level of I_3_(0.8 IW/CPE) increased the build-up of C_mic_ to the tune of 45.73% and 16.73% over I_1_(0.4 IW/CPE) and I_2_(0.6 IW/CPE), respectively. Similarly, an increase in N_mic_ due to the I_3_(0.8 IW/CPE) moisture regime observed was 34.71% over I_1_ and 8.77% in the case of I_2_(0.6 IW/CPE). The corresponding increase in P_mic_ due to the I_3_(0.8 IW/CPE) level of moisture presence was 34.75% and 9.48%, respectively. Further, there is a significant variation in microbial biomass build-up (C_mic_, N_mic_,and P_mic_) due to the usage of various salinity levels. No doubt, the usage of various salinity levels notably decreases the total C_mic_, N_mic_, and P_mic_ to lower salinity levels. The build-up of the biomass of C_mic_, N_mic_, and P_mic_ was recorded lowest under C_2_(12 dSm^−1^) and highest in the C_0_. The decrease in C_mic_ due to C_2_(12 dSm^−1^) was 41.87% and 18.97% for the C_0(Control)_ and C_1_(6 dSm^−1^), respectively. Similarly, there was a decrease in N_mic_ by 35.02% over the C_0(control)_ and 18.12% in C_1_(6 dSm^−1^),and the corresponding decrease in P_mic_ analyzed was 40.54% and 24.05%, respectively (Table 2).

#### 2.3.2. Effect on Microbial Loss

It is evident that the microbial biomass losses of C_mic_, N_mic_, and P_mic_ are significantly affected because of the application of organic fertilizer, but the microbial losses of C_mic_ and N_mic_ were not found to have a difference by using vermicompost and FYM. C_mic_ loss was observed to be the highest under FYM by 15.90% over the control and 1.08% compared to vermicompost. In the cases of N_mic_ and P_mic_, the highest value was observed when applied to vermicompost. There was an increase in N_mic_ loss due to the usages of vermicompost and the results were found to be 0.95% and 13.50% over the FYM and control, correspondingly. The respective increase in P_mic_ loss was analyzed to be 10.78% and 23.09%. Microbial biomass losses of C_mic_, N_mic_, and P_mic_ occurred due to the different levels of moisture regimes, but losses of C_mic_, N_mic_, and P_mic_ were found to be on par among I_3_(0.8 IW/CPE) and I_2_(0.6 IW/CPE) moisture regimes. C_mic_ loss was highest under the I_3_(0.8 IW/CPE) moisture regime, with further losses of 5.25% and 11.07% compared to theI_2_(0.6 IW/CPE) and I_1_(0.4 IW/CPE) moisture regimes, respectively. In the cases of N_mic_ and P_mic_, the highest value was observed under I_3_(0.8 IW/CPE). The increase in the N_mic_ level due to I_3_(0.8 IW/CPE) in the pooled mean was 6.36% over I_2_(0.6 IW/CPE) and 2.08% over the I_1_(0.4 IW/CPE) moisture regime. The corresponding increase in P_mic_ was 4.27% and 0.26%, respectively. Soil microbial biomass losses of C_mic_, N_mic_, and P_mic_ tend to decrease significantly with increasing salinity. Lower microbial biomass losses of C_mic_, N_mic_, and P_mic_ were recorded with C_2_(12 dSm^−1^) compared with the rest of the treatments. There was a decrease in the C_mic_ value by 28.43% and 54.49%, respectively. Correspondingly, the losses in N_mic_ through C_2_(12 dSm^−1^) were 18.35% compared to C_1_(6 dSm^−1^) and 55.80% in the C_0_(control). The corresponding decreases in the P_mic_ recorded were 17.48% and 53.35%, respectively (Table 2).

#### 2.3.3. Effect on Microbial Turnover

Different organic manure treatments significantly affected the turnover of microbial C_mic_, N_mic_, and P_mic_. The minimum microbial turnover was observed under the application of vermicompost by 24.24% in C_mic_, 20.89% in N_mic_, and 10.89% in P_mic_ over the control. It was also observed that there was a decrease in the turnover by 7.89% in C_mic_, 7.24% in N_mic_, and 1.45% in P_mic_ after the application of the FYM treatment. Due to the use of various levels of moisture regimes, the turnover of microbial C_mic_, N_mic_, and P_mic_ was affected. The minimum turnover was noted under the I_3_(0.8 IW/CPE) moisture regime, which was 31.29% in C_mic_, 31.72% in N_mic_, and 30.78% in P_mic_ over the I_1_(0.4 IW/CPE) moisture regime, and there was also a decrease in turnover due to application of the I_2_(0.6 IW/CPE) moisture regime by 11.05%, 2.26%, and 8.68% in C_mic_, N_mic_, and P_mic_, respectively. A high concentration of salinity indicates a variation in the turnover of C_mic_, N_mic_, and P_mic_. The turnover rate of the biomass of C_mic_, N_mic_, and P_mic_ were considerably the maximum observed in the C_0(control)_, and the minimum records were under C_2_(12 dSm^−1^). The decrease in C_mic_ due to C_2_(12 dSm^−1^) was 10.28% over the C_0(control)_and 8.09% in the C_1_(6 dSm^−1^) treatment. Similarly, the decrease in N_mic_ and P_mic_ due to the C_2_(12 dSm^−1^) treatment was 14.95% and 9.00% over the C_0(control)_, while, this was found to be11.08% and 4.86% over the C_1_(6 dSm^−1^) treatment.

#### 2.3.4. Effect on the Annual Flux of C_mic_, N_mic_, and P_mic_ on Organic Fertilizer, Moisture, and Salt Concentration

A significant variation in the amount of annual C_mic_, N_mic_, and P_mic_ fluxes through the C_mic_, N_mic_, and P_mic_ under different organic manures was observed during the study (Table 3). C_mic_, N_mic_, and P_mic_ fluxes due to the usage of vermicompost show good results compared to other treatments, such as M_0(control)_ and M_1(FYM)_. After the use of vermicompost, fluxes were recorded to be 14.36% higher than the C_mic_ flux over the M_0(control)_ and M_1(FYM)_. Similarly, the N_mic_ flux was 8.47% and 37.45% higher than M_1(FYM)_ and M_0(control)_, respectively. The subsequent increases in P_mic_ flux were 37.01% and 12.55% in the mean analysis. Further, the cumulated data was found to have significant variations in annual C_mic_, N_mic_, and P_mic_ fluxes compared to C_mic,_ N_mic_, and P_mic_ under different moisture regimes. C_mic_, N_mic_, and P_mic_ fluxes under the application of irrigation in I_3_(0.8 IW/CPE) moisture regimes were significantly superior over the resting level of the moisture regimes I_1_(0.4 IW/CPE) and I_2_(0.6 IW/CPE). The I_3_ moisture regime was observed to be 45.73% and 16.73% higher than the C_mic_ flux over I_1_ and I_2_, respectively. The N_mic_ flux was recorded to be 34.76% and 8.80% higher over I_1_ and I_2_, respectively, due to an increase in the P_mic_ flux in I_3_(0.8 IW/CPE)_._ The levels of the moisture regimes were 34.69% and 9.50% in mean data validation). The annual fluxes of C_mic_, N_mic_, and P_mic_ were affected by the different levels of saline water (Table 2). The analyzed results of the C_mic_ flux under C_2_(12 dSm^−1^) were 41.86% and 18.97% lower than the C_0(Control)_ and C_1_(6 dSm^−1^), subsequently. The N_mic_ flux was observed to be 35% and 18.07%, and in the case of the P_mic_ flux, it was 40.39% and 24.13% lower than the C_0(Control)_ and C_1_(6 dSm^−1^), respectively. Notably, the lower flux amounts of C_mic_ (170.84 kg ha^−1^ yr^−1^), N_mic_ (32.14 kg ha^−1^ yr^−1^), and P_mic_ (13.26 kg ha^−1^ yr^−1^) were found with C_2_(12 dSm^−1^) compared with the higher amounts observed in C_mic_ (242.37 ha^−1^ yr^−1^), N_mic_ (43.49 kg ha^−1^ yr^−1^), and P_mic_ (18.63 kg ha^−1^ yr^−1^) with the C_0(Control)_.

#### 2.3.5. Soil Properties and the Correlation between Soil C_mic_, N_mic_, and P_mic_

Soil microbial C_mic_, N_mic_, and P_mic_ were positively and significantly correlated to available water, organic carbon, saturated hydraulic conductivity (SHC), and yield of grains, but inversely correlated with the pH and electrical conductivity of the soil (Table 3). The experimental outcomes of the regression analysis of the C_mic_, N_mic_, and P_mic_ of the soil as a dependent variable are presented in Table 4. Around 58.10% of the variability in C_mic_ is explained by the soil OC. An additional 34.30% variability is explained by considering the total nitrogen (N) as a second variable in the equation. Inclusion of the total phosphorus (P) could not improve the variability in the C_mic_. Similar to other variables, soil dehydrogenase and alkaline phosphatase enzyme activity improved the prediction value by 5.90%. The simultaneous effect of the soil OC, activities of enzymes, available water, total N, and the total P and pH of the soil accounted for the 99.30% variation of the C_mic_ in the soil. It can be inferred that the OC contents dominate the soil properties and this explained by observing the maximum variation in C_mic_ in the soil of the mustard crop.

Soil N_mic_ was positively related to C_mic_ and P_mic_ in the equations given below:

N_mic_ = 3.393 + 0.154 C_mic_ (R^2^ = 0.979, *p* < 0.001)

N_mic_ = 0.857 + 2.252 P_mic_ (R^2^ = 0.986, *p* ≤ 0.001)

The results reveal a 97.5% and 98.6% variability in N_mic_ due to C_mic_ and P_mic_, respectively, and a unit of C_mic_ and P_mic_; N_mic_ increased by 0.154 and 2.25 µg g^−1^, respectively.

Equations in Table 4 showed SOC as the first variable in multiple regression and 65.50% of the variability in N_mic_. The inclusion of the total nitrogen as the second variable in the regression equation resulted in 30.10% of the variability in N_mic_. The multiple regression equations further indicated the 99.40% difference in N_mic_ was due to the combined effects of OC, total N, total P, dehydrogenase activity, alkaline phosphatase activity, and available water. The inclusion of soil pH as another variable could not improve the predictive value of N_mic_.

Soil P_mic_ was also positively correlated to C_mic_ and N_mic_ according to equations:

P_mic_ = 1.184 + 0.068 C_mic_ (R^2^ = 0.979, *p* < 0.001)

P_mic_ = −0.248 + 0.438 N_mic_ (R^2^ = 0.986, *p* < 0.001)

The above regression equations showed that the 97.90% and 98.60% differences in P_mic_ were attributable due to C_mic_ and N_mic_, respectively.

### 2.4. Mustard Crop Yield

**Figure 2:**
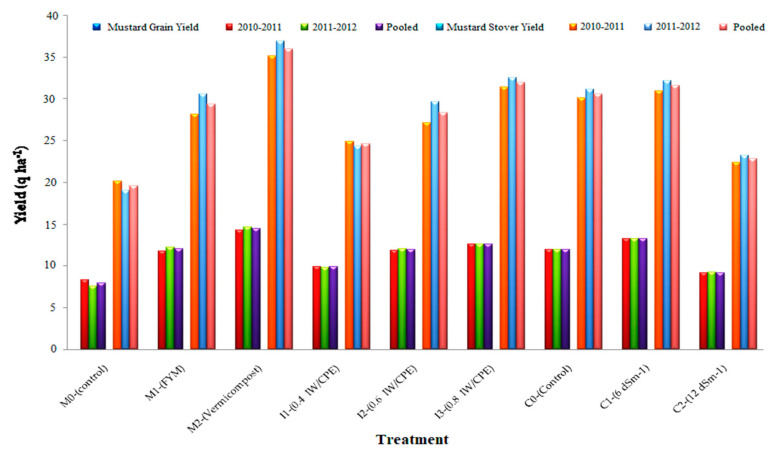
Effect of organic manures, moisture regimes, and salinity levels on mustard grain and stover yield (q ha^−1^) of the crop.

The experimental results of grains and stover yields of the mustard crop were affected by various organic fertilizers, soil moisture regimes, and salinity levels, which are given in Figure 2.

#### 2.4.1. Effect on Grains and Stover Yield

The organic manure treatment (FYM, vermicompost) significantly increases over control grain, and stover yields of the mustard crop (Figure 2). Higher stover and grain yields were recorded due to the usage of vermicompost and were recorded as significantly superior to the FYM and control. The vermicompost increases the pooled grain yield by 82.30% and stover yield by 83.93% above the control. Empanelment in grain yield and stover yield because of FYM were 51.45% and 50.05% over the control, respectively. The moisture regimes significantly increased grain and stover yield and the moisture level regimes differed. The highest grain and stover yields were observed due to the usage of irrigation at I_3_(0.8 IW/CPE) and this moisture regime was significantly higher than the I_2_(0.6 IW/CPE) and I_1_(0.4 IW/CPE) moisture regimes. The I_3_(0.8 IW/CPE) moisture regime increased grain yield by 27.70% and 4.94%, and the stover yield by 30.19% and 12.89% higher than the I_1_(0.4 IW/CPE) and I_2_(0.6 IW/CPE) moisture regimes, respectively. Further, the grain and stover yield of mustard decreases significantly with an increased level of the saline water of C_2_(12 dSm^−1^), but the application of C_1_(6 dSm^−1^) increases grain and stover yield over the control. The application of C_2_(12 dSm^−1^) decreases the total grain yield by 44.75% above the control. The corresponding decrease because of C_1_(6 dSm^−1^) was 30.74% above the C_0(control)_. Decreasing salt concentration levels during both the years and in the pooled mean were C_2_ > C_0_ > C_1_. The application of C_1_(6 dSm^−1^) increases the stover yield non-significantly over the C_0(Control)_. The application of C_2_(12 dSm^−1^) decreases the stover yield by 34.12% and 38.33% over the C_0(Control)_ and C_1_(6 dSm^−1^), respectively.

#### 2.4.2. Grain Yield and Stover Yield Respond with Organic Manures and Moisture Regimes

**Figure 3:**
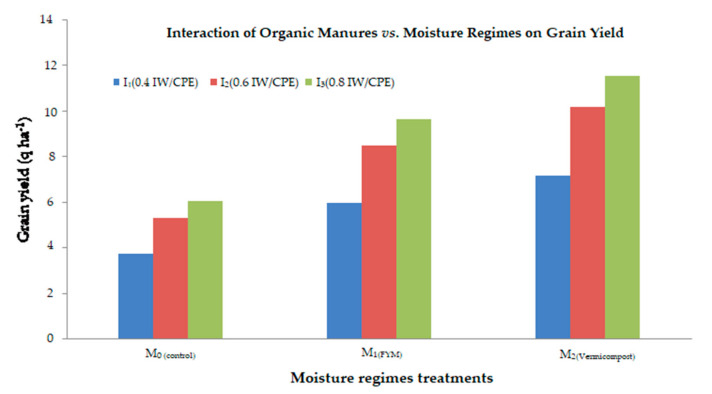
Interaction effect of organic manures and moisture regimes on grain yield (q ha^−1^) of mustard crop.

**Figure 4:**
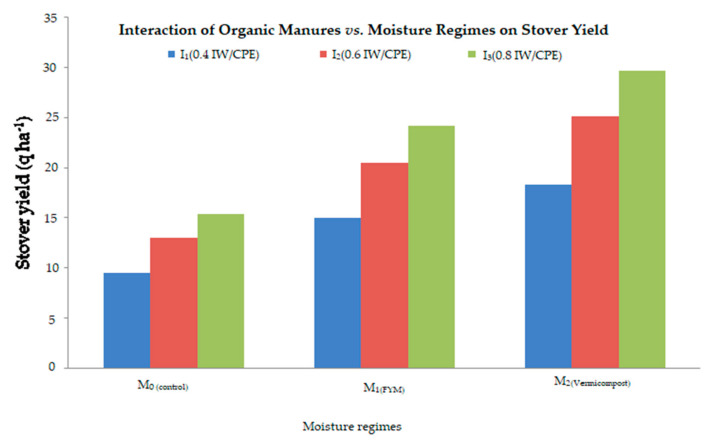
Interaction effect of organic manures and moisture regimes on stover yield (q ha^−1^) of mustard crop.

Grain yield and stover yield response with organic manures and moisture regimes were found to be significant (Figure 3 and Figure 4). Each moisture regime level shows that enhancement is due to the usage of organic fertilizers in the overall pooled mean of both years. Moisture regimes enhance the grain yield above the control irrespective of all organic fertilizers. The enhancement of grain and stover yield with increasing levels of moisture regimes were maximum with the vermicompost followed by the FYM. The highest yield was found due to the combined effect of the I_3_(0.8 IW/CPE) moisture regime and the vermicompost at 5 t ha^−1^, while the minimum was recorded under the I_1_(0.4 IW/CPE) moisture regime with no organic manure (control).

## 3. Discussion

### 3.1. Physico-Chemical Properties of Soil

Soil organic matter contributes to maintaining a desirable physical environment in the soils by affecting and improving the soil’s physical characteristics, expressed through void fraction (or porosity), cluster (or aggregation), bulk density (BD), and moisture-holding capacity [24]. The decomposition of organic matter improves the soil permeability and enhances the water-stable aggregates, which are the result of the synthesis of a complex series of polysaccharides by soil microbes, and the synthesis of new microbial cells and their secretary by-products that contribute to soil-building materials. An enhancement of aggregation and refinement in the soil structure results in an insignificant decline in the mass of the bulk density from the usage of organic fertilizers (Oms), and these can lead to the setting up of low-density materials having intense mineral fractions. The results of this experimentation are in line with an earlier recorded study where bulk density decreases with an increasing amount of organic matter contents in soil [25,26]. Similarly, the effect of different Oms on BD could be well established on hydraulic conductivity (HC) and moisture-holding capacity. The incorporation of Oms significantly increases HC and moisture retention in comparison to the control experiment, and this could be because of a reduction in BD and an enhancement in the soil cluster, which results in increased HC [26]. These findings support the evidence provided that HC correlated positively with the OC contents (r = 0.450 *) of soil. Enhancements in moisture-retention capacity as a result of Oms can be observed from the aggregation, resulting in a favorable pore geometry in the soil [27]. This study is also in line with the existence of a positive correlation between the OC, available moisture content (r = 0.853 *), and HC (r = 0.450 **) in soil [28].

The remarkable decrease in pH is because of the incorporation of organic manures, which leads to an increase in soil EC. The additions of Oms have a positive effect and ascribe the formation of CO_2_ and the organic acids during the process of microbial decomposition towards the counteraction of the negative effects of soil pH. The microbial growth reflected microbial activation, which occurs due to the addition of a substrate amount in the form of organic manures [29]. Carbonic acid (H_2_CO_3_) is formed from the reaction between carbon dioxide, water, and the subsequent reaction with the native calcium carbonate (CaCO_3_) of soil to bring calcium (Ca) into the soil solution. The calcium, due to the influence of microbial decomposition, usually releases sodium (Na) in exchange and reduces the soil pH. This study is in line with other published works indicating the reductions of pH due to the organic materials [30,31]. Besides, these findings show the negative correlation of OC with the pH of the soil (r = −0.383). Electrical conductivity (EC) increases remarkably with the addition of Oms. Due to the usage of farmyard manure (FYM) and vermicompost they are observed to have a maximum EC over the control during the experimentation. In comparison, the maximum value of the EC in FYM and vermicompost added in mustard fields are because of the decomposition of organic matter. After the decomposition of organic materials, the acids are released, which dissolves the saline-sodic salts in the soil solution. The addition of Oms at the time of harvest in the soil increases the CEC significantly. The increase in CEC enhances the root growth of plants, which become a component of soil organic matter after the harvest of the economic parts. These results were supported by previously published studies [22,30,32]. These observations led to a positive and significant correlation of OC with CEC (r = 0.327 *). The enhancement in OC content is directly co-related to manure treatment for the incorporation of organic matter in the soil. The high OC content of soil due to vermicompost could be easily decomposable because of the narrower carbon–nitrogen ratio in comparison to FYM, and this was substantiated due to the subsistence of a remarkable positive correlation between the OC contents [8,9,16].

The application of irrigation water on the bulk density of the soil at various moisture regimes was observed to have an insignificant effect. The saturated hydraulic conductivity (SHC) decreases significantly alongside an increase in the frequency of irrigation. The rhizosphere porosity is directly related to the hydraulic conductivity of soil, and during the study, it has been observed that there is a decrease in saturated hydraulic conductivity at higher moisture regimes. It is also recorded that due to frequent irrigation, there is observed to be enhanced moisture content at 33 kPa and 1500 kPa, leading to a drop-off in the depletion of the profile water and water expense efficacy, and this causes a decrease in SHC. The increase in the frequency of irrigation with the addition of organic carbon causes higher moisture retention. Similarly, it has been recorded that with a maximum frequency of irrigation water, EC increases and pH decreases, which is significantly attributed to the lowering of the proportion of Na in the total salt concentration of soil solution and electroneutral [33,34].

Bulk density shows a non-significant effect due to the application of saline water. SHC increases by increasing the concentration of salt in water and these may contribute to basic changes and directly relates to the change in rhizosphere porosity [8]. It has been reported that soil pH decreases with the enhancement in the ECe of the supplying water [20]. There is a considerable enhancement in the ECe of the soil due to the enhanced levels of saline irrigation water, and with the increasing ECe levels of irrigation water, there is the addition of salt quantity, which results in the higher ECe of the soil.

### 3.2. Soil Microbial Biomass

It is evident that the quantity of C_mic_, N_mic_, and P_mic_ in the microbial biomass increases due to the incorporation of organic materials, leading to the enhancement of the growth of microbes with the inclusion of carbon substrate, which subsequently goes on to decline depending on the quantity of the available carbon contents [35]. This proves that the C_mic_, N_mic_, and P_mic_ were directly correlated with the organic matter of the soil. Vermicompost inclusion enhances the microbial C_mic_, N_mic_, and P_mic_, followed by FYM use as treatment, and shows demarcated results of crop growth at later stages under similar conditions [36]. It has been recorded that the soil microbes actively participate in the biogeochemical processes of soil and play a pivotal role in soil carbon and nitrogen (N) turnover [37,38,39]. This study is in line with other studies that supported the view that the increase in microbial biomass of C_mic_, N_mic_, and P_mic_ is because of scarcity in soil pH and results from the addition of organic contents, and this is supported by the existence of the negative correlation of pH with microbial C_mic_, N_mic_, and P_mic_ [26]. The flux of plant nutrients can be determined by the computation of biomass turnover, and the flux of plant nutrients depends on the pool of nutrients produced from microbial biomass turnover. By using the different types of manure, C_mic_, N_mic_, and P_mic_ turnovers were shown to be higher compared to the control because of the easily metabolized substrate of carbon, resulting in low levels of microbial death in manure soil, leading to the maximum turnover rate in non-manure soil [9,16,18]. It is recommended that the prime source of nutrients are soil microorganisms; however, the total microbial nutrients fluxes are not feasible for plant growth. Due to the nutrient input addition, organic matters via organics bring out seasonal variations in fluxes of microbial nutrients. The microbial biomass of C_mic_, N_mic_ (r = 0.989 **), and P_mic_ (r = 0.989 **) have a significant positive correlation and correlate with the microbial biomass of C_mic_ significantly and positively. The growth and development of the microbial biomass was because of the usage of organic manures through entire crops, and the biomass of C_mic_ with organic carbon (r = 0.762 **) has a significant positive correlation [18,40].

The different moisture regimes because of the application of irrigation show a remarkable increase in microbial C_mic_, N_mic_, and P_mic_. It has been found that the growth of microbes highly increased by keeping the optimum moisture in the soil, as the available water and microbial biomass of C_mic_ (r = 0.890 **), N_mic_ (r = 0.884 **), and P_mic_ (r = 0.869 **) correlate positively and significantly. During the different moisture levels, the microbial C_mic_, N_mic_, and P_mic_ contents were the maximum at higher moisture regimes compared to lower moisture regimes. The microfungal biomass present in soil enhances the microbial biomass of C_mic_, N_mic_, and P_mic_ under the I_3_(0.8 IW/CPE) moisture regime compared to the I_1_(0.4 IW/CPE) and I_2_(0.6 IW/CPE) moisture regimes. Compared to the various moisture regimes, the turnover of microbial biomass was lower under I_3_(0.8 IW/CPE). Therefore, the microbial turnover is directly correlated to the favorable conditions supported by McGill et al. [41]. The microbial biomass of C_mic_ (r = 0.899 **), N_mic_ (r = 0.884 **), and P_mic_ (r = 0.869 **) in the presence of water have a significant positive correlation, which implies the microbial biomass development is dependent on optimum moisture conditions. Soil moisture has a positive influence on microbial biomass, which is reported by Tiemann and Billings [29] and Curtin et al. [35].

The increase in the level of the salinity the of irrigation water leads to a decline in the amount of the microbial biomass of C_mic_, N_mic_, and P_mic_, thereby increasing the concentration of salt during irrigation. During crop growth, the microbial biomass starts to decline and remains the minimum at the harvest stage. The decline in the microbial biomass during crop development and the maximum level of salinity is because of the concentration of salt in water, which badly effects the soil’s physico-chemical properties [42]. It has been reported that the microbial biomass of C_mic_ and N_mic_ decreases with increasing ECe in soil [37]. During the study, it was revealed that in the soil irrigated with saline H_2_O, the total turnover of carbon, nitrogen, and phosphorous decreased by increasing the concentration of salt in water, and this is because of a faster turnover rate under stress conditions. Not only that, but the microbes have used much more energy in microbial metabolism to overcome salt stress [9,18,33].

### 3.3. Crop Yield

The addition of organic fertilizers leads to enhancement in the physico-chemical properties of saline soil and enhances the crop grain and stover yield. Hydraulic conductivity, cation exchange capacity, and water retention show positive results but the other property of soil, viz., bulk density decreased. Improvement in these properties helps to keep available a good amount of plant nutrients and their regular supply during the growth period for the optimum development of crops. The availability of a good amount of nutrients and a suitable environment for nutrient uptake leads to the synthesis of carbohydrates and their efficient cell divisions in various sinks, which brings a significant development in grain yield. Vegetative growth enhances stover yield. The maximum grain and stover yield were obtained by the application of vermicompost at 5 t ha^−1^ over the control and FYM @ 10 t ha^−1^. Vermicompost shows an enhancement over FYM based on circumstantial evidence because vermicompost contains the optimum nutrient C:N ratio and high-status-available nutrients, having hormones that increase the level of enzymes [19]. The humus colloidal complex is a valid reason to bring positive changes in soil properties because it is coupled with the good nutrient content of vermicompost and it directly contributes to the nutrient pool of the soil [16,42]. A higher amount of metabolite production and good photosynthetic efficiency during the vegetative growth via vermicompost ensured the continuous supply and gradual release of nutrients, resulting in a higher grain yield and stover yield because of the translocation of photosynthesis at different sinks [39]. By the application of various organic materials, higher grain and stover yields were obtained and were positively correlated, significant of the mean grain yield with the organic carbon contents of the soil (r = 0.661 **), and the microbial biomass of C (r = 0.883 **), N (r = 0.898 **), and P (r = 0.877 **).

The higher stover and grain yield were produced by irrigation in the I_3_(0.8 IW/CPE) moisture regime. An enhancement in photosynthesis occurred during plant development, which is due to proper soil moisture, and affected the growth and development of the plant, and the results were maximum towards storage sites. An assimilation of plant sinks at a later phase, viz., sexual structures, enhanced the movement of storage compounds to respective sites, which happened by increasing moisture in the root zone, leading to an increase in crop yield. Growth inhibition occurred due to salt concentration irrespective of these low irrigations, aggravating water stress. A significant and positive correlation exists between available water (r = 0.794 **) and grain yield. Ghatak et al. [40] found the maximum grain yield because of the usage of three irrigations at branching, flowering, and grain development stages to the extent of 26.50%, 12.08%, and 54.96% over two, one or no irrigation, respectively. Kumawat [30] recorded three irrigations at flowering, branching, and siliqua development stages that significantly enhanced functional yields above the first irrigation at flowering and the next two irrigations at siliqua development and flowering.

The consecutive irrigations significantly increased the stover yield. This enhancement leads to an increase in the availability of moisture, which led to a suitable nutritional environment during the different growth stages of the crop. This results in a good amount of nutrient uptake, which helps in promoting the functioning of the protoplasm, meristematic activities, larger cell sizes, and their formations that help in crop growth and yield [32].

The salinity level greatly affected the yield of grains and the stover yield. ECe 6 dSm^−1^ enhances the grain yield compared to the control but shows growth retardation at ECe 12 dSm^−1^. The low concentration of salinity enhances the yields because of the use of salt constituents as nutrients during metabolic activities; however, there is low yield when salinity increases because of increases in the osmotic pressure of the soil solution.

Irrigating with saline water leads to enhancing the Cl^−^ and SO_4_^2−^ of the Na, Ca, and Mg in the soil that affects the plant growth development due to high osmotic stress, scarcity of water for physiological activity, and toxic effects towards important ions. Researchers have found that by increasing the level of the salt concentration in water, they can enhance the EC of soil and minimize the availability of N, P, and K [22,26,38]. The availability of nutrients in the soil is affected by salinity via modifying retention, fixation, and transformation of nutrients in soils, changing the absorption of nutrients that affect the growth and development of plants [7,43]. The high concentration of salts also affects the enzyme behavior and application of photosynthates in plants. Several researchers found that cationic (Ca, Mg, Na, K) inequality affects the rate of photosynthesis and the activity of stroma enzymes [7,44]. Due to the influence of salt, there is an enhancement in the EC of soil that results from a significant decrease in yield, which overall results in the reduction in grain and stover yield by affecting the availability of water and nutrients for the plants. It is similar to the studies that reported the negative correlation between the ECe (r = 0.112) of soil and grain yield [23,30,34,45].

## 4. Materials and Methods

### 4.1. Experimental Site

**Figure 5:**
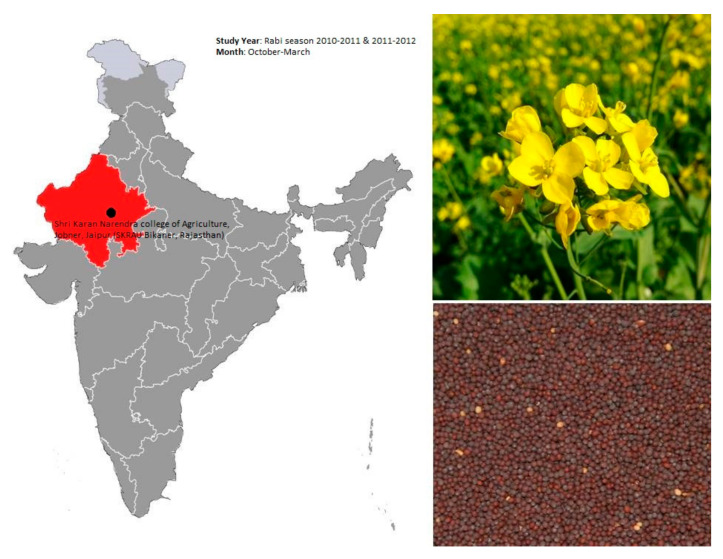
Experimental site and growing period of mustard (*Brassica juncea* (L.) Czern.) crop in arid region.

Experiments were conducted in the rabi season. Data presented is pooled data of two years, and the research was conducted at the experimental farm of Agriculture College of Shri Karan Narendra, Jobner, Jaipur (SKRAU Bikaner, Rajasthan, India), located 45 km west of Jaipur district (26°58′14.16′′ N latitude, 75°22′44.76′′ E longitude) between the elevation range of 420–450 m AMSL (Figure 5). The study region is usually tropical arid to semi-arid. The winter temperature ranges from minus −2 to 30 °C, and summer temperatures vary from 25 to 48 °C. The average annual rainfall varies from 0 to 650 mm, and the majority of rainfall is expected in the monsoon period from July to September. The main source of water for irrigation is underground water. The water level is approximately 30–40 m deep in the soil. From the last five years, cropping history indicated mustard as the main rabi crop followed by a few spices and food grain crops, such as methi (*Trigonella foenum-graecum* L.) and barley (*Hordeum vulgare* L.). Soil properties at the trial field are loamy sand soil of family hyperthermic, *Typic ustipsamment*. The experimental soils (0 to 15 cm depth) were taken randomly from each site selected for the trial. The samples of the representative compound were prepared and subjected to physical, chemical, and mechanical evaluation. These were dried, sieved (2 mm), and properly kept at 4 °C, and used for the analysis of experimental activities (Table 5).

### 4.2. Experimental Observation

The study was carried out by designing a split-plot with 27 treatment combinations having three replicates of each 3 forms of bio-based manures (control, farmyard manure at 10 t ha^−1^, and vermicompost @ 5 t ha^−1^) and moisture regimes (0.4, 0.6, 0.8 IW/CPE ratio) at the experimental site, and saline irrigation water applied (control, 6, 12 dSm^−1^) in sub-plot. The soil samples (0 to 15 cm) were drawn to analyze the properties of soil during the harvest period of the mustard crop. Undisturbed samples of soil were obtained by using cores (the core diameter is 7 cm and the length is 8 cm) to determine the bulk density [46]. By the constant head method of Klute and Dirksen [47] was used for obtaining saturated hydraulic conductivity (SHC) of soil from undistributed soil. Soil pH, CEC, and ECe were determined by using the standard method. Analysis of organic carbon (OC) was done by Walkley and Black’s [48] method, where chromic acid gets oxidized with the organic matter and un-decomposed K_2_Cr_2_O_7_ titrated with {(NH_4_)_2_Fe (SO_4_)_2_·6H_2_O)}. Soil moisture retention was determined by using the apparatus of pressure plate at 33 kPa and 1500 kPa tensions, as explained by Gardner [49], and the amount of water available was measured at harvest time when the moisture retention difference was 33 kPa and 1500 kPa. The pre-conditioned soils were fumigated by ethanol-free chloroform (CHCl_3_) [50,51]. By repeated extraction and evacuation with 0.5 M Potassium sulphate (1:4) for half-hour, CHCl_3_ was removed from soil samples. By the same process, non-fumigated soil samples were extracted. Soil extract organic carbon was determined by using Vance et al. [52] acid dichromate method. C_mic_ was assessed as biomass carbon, BC = EC/0.45 and extractable carbon (EC) is determined from fumigated and non-fumigated treatments of carbon extracted. N_mic_ was also assessed by the CHCl_3_ fumigation procedure using K_2_SO_4_ extract [46]_._ The amount of nitrogen (N)of the soil was extracted by using the Kjeldahl digestion method. N_mic_ was analyzed as BN = EN/0.54, extractable nitrogen (EN) was equivalent to obtained from fumigated and non-fumigated samples [50]. Measurement of P_mic_ was carried out as per Brookes et al. [53] and fumigation was done similarly as that of C_mic_ estimation. P_mic_ was dependent on the variation between non-fumigated and fumigated samples. Inorganic-P (Pi) was determined as proposed by Olsen et al. [54]. P_mic_ was calculated as the difference of CHCl_3_ release Pi with a KP value of 0.40 predicting 40% Pi in the microbial biomass of soil sample. Turnover of microbial biomass was assessed as proposed by McGill et al. [41]. Microbial turnover was assessed from the total calculated losses at times of sampling and the average present (build-up) totality of microbial biomass. Loss values were recorded as negative variations in microbial biomass between the collections of sampling months. The annual flux (C, N, P) of microbial biomass were computed by formula proposed by Brookes et al. [53] and treated with a turnover time of 1.25 years [51]. C, N, and P flux (kg ha^−1^ yr^−1^) = biomass C, N, or P (kg ha^−1^)/turnover time (1.25).

### 4.3. Irrigation Water

The irrigation water was applied into the experimental field for study by using a graduated volumetrically cylindrical water reservoir through PVC hosepipe, and the flow rate of irrigation water was regulated through control on the basis of a value keeping 50 mm of irrigation and used as when total pan evaporation equaled to 125, 83.3, and 62.5 mm for 0.4, 0.6, and 0.8 IW/CPE, alternatively. The irrigation water of various salinity levels was synthesized by dissolving required quantities at NaCl, Na_2_SO_4_, NaHCO_3_, CaCl_2_, and MgCl_2_ in base water maintained with the ratio of Na:Ca:Mg as 60:25:15 and Cl:SO_4_:HCO_3_ as 2:1:1. Each experimental plot was separated by a one meter non-experimental buffer space to check seepage from the adjacent plots.

### 4.4. Field Preparation and Crop Management

The research area was prepared through disc plough and cross harrowing, and leveled with the laser to make the field a well-usable condition 15 days before the trial of the experiment, which was then followed by the design of the trial. Mustard (*Brassica juncea*) variety Pusa Jaikishan (Bio-902) was used as the test crop for the experiment. The grains of the selected variety in the first year were sown at 3 kg ha^−1^ by the pora method in October 2010 and the month of November 2011 in the next year. In both consecutive years, the proportion of nitrogen (N) at 15 kg ha^−1^, potash (K_2_O) at 30 kg ha^−1^, and phosphorus (P_2_O_5_) at 40 kg ha^−1^ were obtained from Urea, Diammonium Phosphate (DAP), and Muriate of Potash (MOP). The basal fertilizers were placed 9 cm deep with the help of a fertilizer drill before grains sowing, and nitrogen proportion was obtained from Urea fertilizer in three topdressings at 30 day intervals.

### 4.5. Statistical Analysis

#### 4.5.1. Variance and Test of Significance

Analysis of the significance of variation in research data assess from various trial effects, and Fisher’s test [55] was used for statistical analysis of data. The test of significance was determined by critical differences and was found significant at 5% probability. To elucidate the nature and the magnitude of treatment effects, data with SEm± and CD (*p* = 0.05) were prepared.

#### 4.5.2. Correlation

Multiple regression equations were used to study the affinity of soil C_mic_, N_mic_, P_mic_, and other properties of soil. Gomez and Gomez [56] method was used for statistical analysis.

## 5. Conclusions

Our study indicated that the interactive outcome of a soil water regime and the application of organic manures in the form of farmyard fertilizers and vermicompost significantly influenced the mustard grain yield and stover yield. The maximum grain and stover yield recorded was due to the application of irrigation at I_3_(0.8 IW/CPE). The application of vermicompost @ 5 t ha^−1^ increases moisture retention at 33 kPa and 1500 kPa in an arid environment. The irrigation at I_3_(0.8 IW/CPE) increased the pooled grain yield by 27.70% and 4.94% over the I_2_(0.6 IW/CPE) and I_1_(0.4 IW/CPE) moisture regimes, respectively. The organic treatment recorded was significantly superior to FYM and increases the pooled grain yield and stover yield by 82.30% and 83.93% over the control, respectively. The soil microbial biomass (C_mic_, N_mic_, and P_mic_ contents) increases significantly in the mustard grain field in an arid climate. The microbial biomass build-up increased by 43.13% over the control and 14.36% over the farmyard manures. Various factors such as climate variables, soil types, saline water concentration, and soil moisture regime affect the growth of the microbial biomass. The high concentration of salinity indicates a variation in the turnover of C_mic_, N_mic_, and P_mic._ The use of vermicompost was recorded to be 14.36% higher than the C_mic_ flux over the M_0(control)_ and M_1(FYM)_. The soil organic manure availability and the continuous process of acidification were the key drivers of soil microbial biomass and activity changes during the mustard planting. Therefore, our study is helpful for understanding the characteristics of the stoichiometric homeostasis of the soil microbial biomass of carbon, nitrogen, and phosphorus in the arid climate of India. Our investigation also indicated that the SHC of the soil was enhanced by the application of farmyard fertilizers and vermicompost. The SHC of the soil significantly increases by 3.50% and 5.76% over control. The bulk density and soil pH decrease significantly in mustard field, while the OC, CEC, and ECe of the soil increase. Therefore, our study concludes that the application of organic manures and moisture regimes leads to the enhancement of the population of microorganisms and crop production. The soil biological activity, such as the microbial biomass of C_mic_, N_mic_, and P_mic_, their build-up, losses, fluxes, and the turn-over rate, reduces due to application of increasing levels of saline water. Therefore, this study highlights the need for sustainable soil management that can facilitate the formation of soil aggregates. The addition of organic manure and the frequent irrigation of water is more efficient in improving soil’s physical and chemical properties.

## Figures and Tables

**Table 1:** Effect of organic manures and moisture regimes on physico-chemical properties of soil at harvest.

Treatments	BD (Mg m^−3^)	SHC (cm h^−1^)	33 kPa (%)	1500 kPa (%)	Available Water (%)	pH	EC (dSm^−1^)	CEC (cmol(P+)kg^−1^)	OC (g kg^−1^)
**Organic manures**									
M_0_(control)	1.62	7.11	10.70	3.68	7.02	8.58	2.60	5.12	2.00
M_1_(FYM)	1.52	9.18	12.16	3.87	8.29	8.22	2.61	5.27	2.45
M_2_(vermicompost)	1.49	8.39	12.27	3.91	8.36	7.82	2.64	6.10	2.57
SEm±	0.02	0.14	0.15	0.03	0.06	0.13	0.03	0.06	0.03
CD (*p* = 0.05)	0.05	0.41	0.44	0.08	0.18	0.37	0.08	0.17	0.09
**Moisture regimes**									
I_1_(0.4 IW/CPE)	1.53	8.71	11.23	3.72	7.52	8.83	2.58	5.47	2.30
I_2_(0.6 IW/CPE)	1.54	8.06	11.28	3.84	7.44	8.12	2.60	5.50	2.29
I_3_(0.8 IW/CPE)	1.55	7.90	12.62	3.91	8.72	7.67	2.66	5.51	2.43
SEm±	0.02	0.14	0.15	0.03	0.06	0.13	0.03	0.06	0.03
CD (*p* = 0.05)	NS	0.41	0.44	0.08	0.18	0.37	0.08	NS	0.09
**Salinity levels**									
C_0_(control)	1.57	7.98	12.63	4.13	8.50	8.61	2.42	5.52	2.58
C_1_(6 dSm^−1^)	1.54	8.26	11.62	3.75	7.87	8.16	2.59	5.49	2.45
C_2_(12 dSm^−1^)	1.52	8.44	10.89	3.59	7.30	7.85	2.84	5.48	2.00
SEm±	0.02	0.13	0.14	0.03	0.08	0.12	0.04	0.07	0.03
CD (*p* = 0.05)	NS	0.38	0.40	0.09	0.22	0.33	0.10	NS	0.09

BD—bulk density, SHC—saturated hydraulic conductivity, EC—electrical conductivity, CEC—cation exchange capacity, OC—organic carbon, NS—not significant.

**Table 2:** Effect of organic manures, moisture regimes, and salinity levels on build-up, loss, turnover and annual flux rate of soil microbial biomass C, N, and P.

Treatments	Build-Up (µg g^−1^)			Loss (µg g^−1^)			Turnover (yr^−1^)			Flux (kg ha^−1^ yr^−1^)		
	C	N	P	C	N	P	C	N	P	C	N	P
**Organic manures**												
M_0_(control)	93.40	17.39	7.52	49.22	7.48	3.42	0.533	0.434	0.533	167.38	31.16	13.48
M_1_(FYM)	116.90	22.03	9.15	57.05	8.41	3.80	0.494	0.385	0.494	209.49	39.49	16.41
M_2_(Vermicompost)	133.69	23.90	10.31	56.44	8.49	4.21	0.429	0.359	0.429	239.58	42.83	18.47
Sem±	2.35	0.31	0.14	1.16	0.16	0.10	0.007	0.005	0.007	3.81	0.68	0.28
CD (*p* = 0.05)	6.75	0.88	0.40	3.34	0.46	0.28	0.020	0.015	0.020	10.93	1.94	0.80
**Moisture regimes**												
I_1_(0.4 IW/CPE)	92.83	17.66	7.54	51.39	8.18	3.74	0.558	0.465	0.558	166.35	31.64	13.52
I_2_(0.6 IW/CPE)	115.89	21.87	9.28	54.23	7.85	3.84	0.472	0.361	0.472	207.68	39.19	16.63
I_3_(0.8IW/CPE)	135.28	23.79	10.16	57.08	8.35	3.85	0.425	0.353	0.425	242.43	42.64	18.21
Sem±	2.35	0.31	0.14	1.16	0.16	0.10	0.007	0.005	0.007	3.81	0.68	0.28
CD (*p* = 0.05)	6.75	0.88	0.40	3.34	0.46	0.28	0.020	0.015	0.020	10.93	1.94	0.80
**Salinity levels**												
C_0_(Control)	135.25	24.21	10.40	66.15	9.80	4.57	0.504	0.415	0.504	242.37	43.39	18.63
C_1_(6 dSm^−1^)	113.42	21.18	9.18	54.29	8.28	3.89	0.494	0.401	0.494	203.25	37.95	16.46
C_2_(12 dSm^−1^)	95.33	17.93	7.40	42.27	6.29	2.98	0.457	0.361	0.457	170.84	32.14	13.26
Sem±	2.84	0.37	0.18	1.30	0.20	0.11	0.009	0.007	0.009	4.54	0.66	0.33
CD (*p* = 0.05)	7.98	1.04	0.51	3.64	0.55	0.31	0.025	0.018	0.025	12.76	1.85	0.93

**Table 3:** Correlation coefficient ® between soil C_mic_, N_mic_, and P_mic_ and other soil properties.

	C_mic_	N_mic_	P_mic_	OC	CEC	EC	pH	SHC	Available Water
C_mic_	1.000	0.989 **	0.989 **	0.762 **	0.544 **	−0.294	−0.695 **	0.026	0.899 **
N_mic_		1.000	0.993 **	0.809 **	0.527 **	−0.286	−0.707 **	0.105	0.884 **
P_mic_			1.000	0.802 **	0.544 **	−0.312	−0.666 **	0.034	0.869 **
OC				1.000	0.327	−0.347	−0.383 *	0.450 *	0.853 **
CEC					1.000	0.028	−0.650 **	0.221	0.443 *
EC						1.000	0.041	0.108	−0.243
pH							1.000	−0.262	−0.572 **
SHC								1.000	0.279
Available water									1.000

* Significant at 5% level of significance. ** Significant at 1% level of significance. SHC—saturated hydraulic conductivity, EC—electrical conductivity, CEC—cation exchange capacity, OC—organic carbon

**Table 4:** Effect of soil properties on predictability of microbial biomass C, N, and P in soil.

Microbial Biomass	Regression Equation	Coefficient of Determination (R^2^)
C_mic_	−32.388 + 62.889 OC	0.581 **
	−38.298 + 29.630 OC + 4122.916 Total-N	0.924 **
	−38.784 + 43.284 OC + 5932.753 Total-N − 2264.053 Total-P	0.931 **
	−97.253 − 67.858 OC − 5892.055 Total-N + 4429.210 Total-P + 23.735 DHA	0.958 **
	−69.697 + 24.025 OC + 382.839 Total-N – 305.447 Total-P − 0.104 DHA + 13.073 APA	0.990 **
	−68.281 + 23.927 OC + 359.099 Total-N − 320.123 Total-P − 0.026 DHA − 13.062 APA − 0.159 pH	0.990 **
	−24.994 + 59.680 OC + 762.708 Total-N − 1306.141 Total-P − 5.861 DHA + 20.548 APA − 2.814 pH − 8.982 Available water	0.993 **
N_mic_	−3.262 + 10.422 OC	0.655 **
	−4.124 + 5.549 OC + 604.010 Total-N	0.956 **
	−4.133 + 5.707 OC + 625.009 Total-N − 26.270 Total-P	0.956 **
	−11.263 − 7.845 OC − 816.939 Total-N + 789.925 Total-P + 2.894 DHA	0.973 **
	−8.937 − 0.089 OC − 287.258 Total-N + 390.259 Total-P + 0.882 DHA + 1.104 APA	0.982 **
	−13.427 + 0.221 OC − 211.991 Total-N + 436.791 Total-P + 0.636 DHA + 1.140 APA + 0.505 pH	0.983 **
	−0.999 + 10.485 OC − 96.113 Total-N + 153.700 Total-P − 1.040 DHA +3.290 APA − 0.257 pH − 2.579 Available water	0.994 **
P_mic_	−1.664 + 4.558 OC	0.644 **
	−2.046 + 2.405 OC + 266.840 Total-N	0.947 **
	−2.091 + 3.656 OC + 432.637 Total-N − 207.408 Total-P	0.959 **
	−5.047 − 1.964 OC − 165.308 Total-N + 131.051 Total-P + 1.200 DHA	0.973 **
	−4.314 + 0.480 OC + 1.625 Total-N + 5.093 Total-P + 0.566 DHA + 0.348 APA	0.978 **
	−6.015 + 0.598 OC + 30.144 Total-N + 22.724 Total-P + 0.473 DHA + 0.362 APA + 0.191 pH	0.979 **
	−1.321 + 4.475 OC + 73.912 Total-N − 84.200 Total-P − 0.160 DHA + 1.173 APA − 0.096 pH − 0.974 Available water	0.986 **

** Significant at 1% level of significance.

**Table 5:** Basic soil (0–15 cm) physico-chemical characteristics of experiment field.

Soil Characteristics	Values
**Mechanical composition**	
Coarse sand (%)	25.30
Fine sand (%)	57.40
Silt (%)	9.50
Clay (%)	7.50
Textural class	Loamy sand
**Physical properties**	
Bulk density (Mg m^−3^)	1.52
Particle density (Mg m^−3^)	2.52
**Chemical properties**	
pH	8.50
ECe (dS m^−1^) at 25 °C	2.54
CEC [cmol(p^+^) kg^−1^]	5.15
Exchangeable Na [cmol(p^+^) kg^−1^]	1.08
ESP	20.97
CaCO_3_ (g kg^−1^)	16.08
**Soluble cations (mmol L^−1^)**	
Na^+^	22.60
Ca^2+^ + Mg^2+^	2.40
K^+^	0.20
Soluble anions (mmol L^−1^)	
CO_3_^2−^ + HCO_3_^−^	6.40
Cl^−^	8.70
SO_4_^2−^	10.10
Organic carbon (g kg^−1^)	1.80
Available N (kg ha^−1^)	133.60
Available P (kg ha^−1^)	9.48
Available K (kg ha^−1^)	159.15

## Data Availability

Data is contained within the article.
